# Four Novel Prognostic Genes Related to Prostate Cancer Identified Using Co-expression Structure Network Analysis

**DOI:** 10.3389/fgene.2021.584164

**Published:** 2021-04-01

**Authors:** Tao Feng, Dechao Wei, Qiankun Li, Xiaobing Yang, Yili Han, Yong Luo, Yongguang Jiang

**Affiliations:** Department of Urology, Beijing Anzhen Hospital, Capital Medical University, Beijing, China

**Keywords:** prostate cancer, biomarker, weight co-expression network analysis, gene set enrichment analysis, gene set variation analysis, small molecular drugs

## Abstract

Prostate cancer (PCa) is one of the most common malignancies for males, but very little is known about its pathogenesis. This study aimed to identify novel biomarkers associated with PCa prognosis and elucidate the underlying molecular mechanism. First, The Cancer Genome Atlas (TCGA) RNA-sequencing data were utilized to identify differentially expressed genes (DEGs) between tumor and normal samples. The DEGs were then applied to construct a co-expression and mined using structure network analysis. The magenta module that was highly related to the Gleason score (*r* = 0.46, *p* = 3e–26) and tumor stage (*r* = 0.38, *p* = 2e–17) was screened. Subsequently, all genes of the magenta module underwent function annotation. From the key module, CCNA2, CKAP2L, NCAPG, and NUSAP1 were chosen as the four candidate genes. Finally, internal (TCGA) and external data sets (GSE32571, GSE70770, and GSE141551) were combined to validate and predict the value of real hub genes. The results show that the above genes are up-regulated in PCa samples, and higher expression levels show significant association with higher Gleason scores and tumor T stage. Moreover, receiver operating characteristic curve and survival analysis validate the excellent value of hub genes in PCa progression and prognosis. In addition, the protein levels of these four genes also remain higher in tumor tissues when compared with normal tissues. Gene set enrichment analysis and gene set variation analysis for a single gene reveal the close relation with cell proliferation. Meanwhile, 11 small molecular drugs that have the potential to treat PCa were also screened. In conclusion, our research identified four potential prognostic genes and several candidate molecular drugs for treating PCa.

## Introduction

Prostate cancer (PCa) is the second most frequent malignancy and the fifth leading cause of death in males throughout the world (Bray et al., 2018). Currently, prostate biopsy has become the standard for diagnosing PCa worldwide. Meanwhile, prostate-specific antigens (PSA) are considered a reliable prostate tumor marker, especially in the early stages of PCa. Despite the wide use of PSA tests in screening for PCa, this approach has some restrictions. In several non-malignant cases, such as those with prostatitis and benign prostatic hyperplasia, serum PSA frequently increases, affecting the accuracy of the PSA test (McDonald et al., 2014). In addition, many patients with widespread metastases from PCa showed poor differentiation or have neuroendocrinal differentiation on histology with typically low PSA levels (Caram et al., 2016). Moreover, physicians and patients often overestimate the abilities of PSA testing, which leads to overdiagnosis and overtreatment of indolent PCa (Ciatto et al., 2000; Dall’Era et al., 2008; Bryant et al., 2012). Furthermore, there are no clear serum PSA levels that assist in assessing a patient with PCa. On the other hand, it is well known that PCa is a serious threat to the health of males, especially those with advanced stage, drug resistance, neoplasm recurrence, and tumor metastasis, always contributing to death even after combined treatment. Therefore, a novel and specific biomarker for PCa needs to be explored.

Since the advent of microarray and high-throughput sequencing technology, bioinformatics has played a significant role in many fields, especially in the medical field (Gu and Chen, 2014; Li et al., 2014; Huang and Huang, 2015; Turei et al., 2015). In recent years, more potential biomarkers have been discovered. However, the vast majority of studies focus only on the differences in expression between different samples, and interactions among the genes have been largely neglected (Song et al., 2018; Pudova et al., 2019; Zheng et al., 2019).

To further investigate the underlying connection and relative importance of each gene, we applied structure network algorithms, which identify function-specific modules based on network topological importance (Bu et al., 2020). A weighted gene correlation network analysis (WGCNA) was constructed, and genes with similar expression profiles were clustered into the same module. Then, the correlation between the module and clinical phenotype was analyzed to choose the module that is most significantly related to the clinical disease phenotype. Gene Ontology (GO) and Kyoto Encyclopedia of Genes and Genomes (KEGG) analyses of genes within the key module were conducted to explore the potential functions. After a series of screenings, four hub genes (CCNA2, CKAP2L, NCAPG, and NUSAP1) that could truly predict the progression and prognosis of PCa were found in our study. Gene set enrichment analysis (GSEA), and gene set variation analysis (GSVA) were used to investigate potential biological functions. Meanwhile, a variety of databases, such as Gene Expression Omnibus (GEO), The Cancer Genome Atlas (TCGA), and HPA, were utilized to verify the genes in different methods, such as survival analysis or receiver operating characteristic (ROC) curve. Also, TIMER was applied to explore the immune infiltration of the hub genes. In addition, cBioPortal was used to assess genetic alterations of those four genes. Finally, the Connectivity map (CMap) was used to further screen the correlation of small molecule drug targets.

## Materials and Methods

### Data Acquisition and Study Design

**FIGURE 1:**
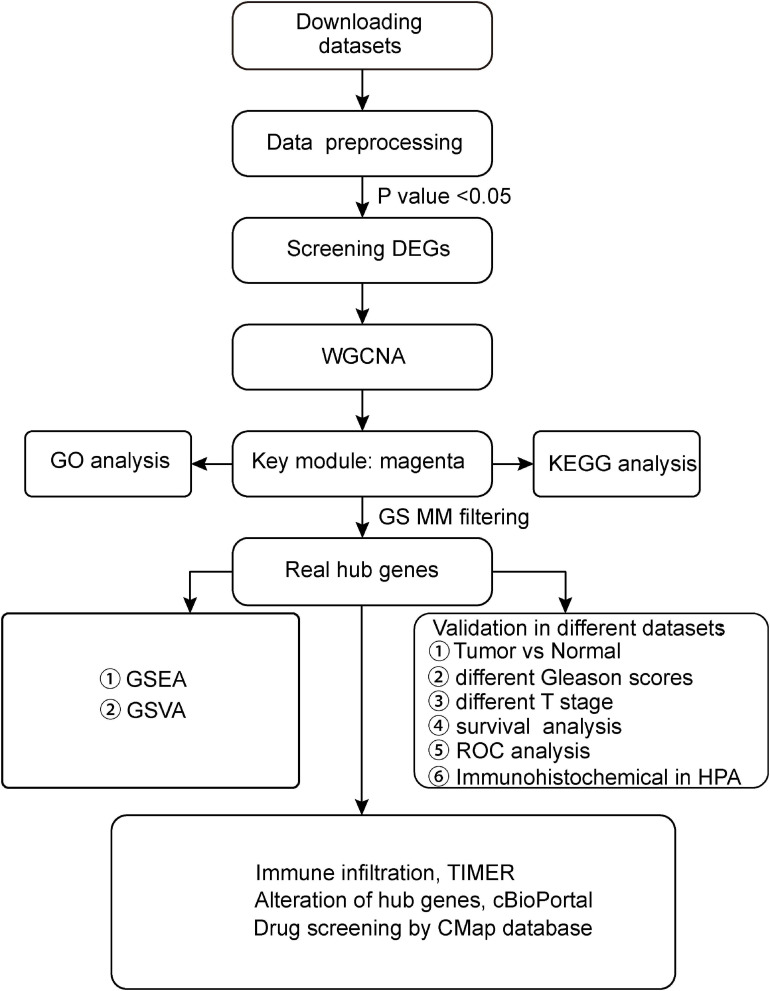
Flow chart of our study. GEO, Gene Expression Omnibus; TCGA, The Cancer Genome Atlas; GO, Gene Ontology; KEGG, Kyoto Encyclopedia of Genes and Genomes; WGCNA, Weighted Gene Co-expression Network Analysis; GS, gene significance; MM, module membership; TNM, Tumor Node Metastasis; GSEA, Gene Set Enrichment Analysis; and GSVA, Gene Set Variation Analysis.

The RNA-sequencing (RNA-seq) and clinical information for PCa were acquired from the TCGA database^1^, which included 498 PCa samples and 52 normal prostate samples. The data sets of TCGA were used to screen differentially expressed genes (DEGs); perform WGCNA; verify the hub genes; and perform GSEA, GSVA, and survival analysis in our study. Simultaneously, all microarray data sets and other information were downloaded from the GEO database of the NCBI databases^2^. The data set GSE32571, which was obtained from the Affymetrix Human Gene 1.0 ST, was used to verify the expression of hub genes between normal and tumor samples. The GSE70770 was used for expression profiling based on GPL10558 (Illumina HumanHT-12 V4.0) and comprised 207 tumor samples. The data set was utilized to explore the expression of hub genes with different Gleason scores and stages. Finally, another data set of GSE141551, which was obtained on the Illumina Human HT-12 WG-DASL V4.0, was used as a testing set to further verify our results. The cohorts that did not undergo WGCNA analysis were selected as internal training validation sets, and the other data sets that have undergone WGCNA analysis were used as external validation data sets. Therefore, TCGA was chosen as the training and internal validation data sets, whereas GSE32571, GSE70770, and GSE141551 were used as external validation data sets. Detailed information on these data sets is shown in Table 1, and the workflow of our research is presented in Figure 1.

**TABLE 1:** Information on data sets in our study.

**Data sets**	**TCGA**	**GSE32571**	**GSE70770**	**GSE141551**
**Platform**	**Training validation data sets**		**External validation data sets**	

	**Illumina RNASeqV2**	**Affymetrix Human gene 1.0 ST**	**Illumina HumanHT -12 V4.0**	**Illumina HumanHT-12 WG-DASL V4.0**

GPL ID	–	GPL6947	GPL10558	GPL14951
**Summary**				
Total	550	98	207	503
Prostate cancer	498	59	207	503
Normal prostate	52	39	–	–
**Gleason score**				
4	–	–	–	10
5	–	–	2	–
5∼6	–	–	–	229
6	45	–	35	–
7	249	–	142	–
7 = 3 + 4	–	–	–	184
7 = 4 + 3	–	–	–	40
8	59	–	13	–
9	133	–	10	–
10	4	–	1	–
8∼10	–	–	–	40
Unknown	–	–	4	–
**T stage**				
T1	–	–	103	–
T2	187	–	73	–
T3	286	–	25	–
T4	10	–	–	–
Unknown	15	–	6	–

### Data Preprocessing and DEG Screening

All microarray data of GEO data sets were subjected to quality control, background correction, logarithmic conversion, and removal of batch effects processing, using the R package “limma” (Ritchie et al., 2015). The samples without clinical data were then excluded from subsequent analysis. The RNA-seq data of TCGA were normalized with the “DESeq2” R package. The three R packages—DESeq2, analyzed based on a negative binomial distribution method (Love et al., 2014); limma, based on linear models and empirical Bayes methods (Ritchie et al., 2015); and edgeR, based on an overdispersed Poisson model (Robinson et al., 2009)—were then utilized to screen the DEGs between normal and cancer samples. Finally, the overlapping DEGs by adjusting *P* < 0.05 were considered as target genes and further analyzed.

### WGCNA

The DEGs were constructed based on a weighted gene co-expression using the R package “WGCNA” (Langfelder and Horvath, 2008). First, the function “goodSamplesGenes” in the “WGCNA” package was used in maintaining the RNA-seq data from overlapping DEGs if they proved to be good samples. Then, the outlier samples were removed to ensure that the results of network construction are more reliable. Second, a matrix of similarity was constructed by Pearson’s correlation analysis of all pairs of genes. After that, the matrix was performed to construct a scale-free co-expression network based on an optimal soft threshold power β (Chen et al., 2018). A similar matrix was then transformed into a topological overlap matrix (TOM). This TOM could measure the network connectivity of a gene, which was defined as the sum of its adjacency to all other network-generated genes (Botia et al., 2017). At the same time, the average linkage of hierarchical clustering was analyzed by the TOM-based dissimilarity measure with a minimum gene group size of 50 for the gene dendrogram. The dissimilarity of module eigengenes was calculated to further analyze the module.

### Identify Significant Relevant Module and Module Functional Annotation

The correlation between the modules and the clinical phenotypes was analyzed by the module–trait relationship analysis of WGCNA. Then, the module that is most relevant to the clinical phenotype was found. Here, the magenta module that significantly connects with the Gleason score of PCa was chosen. The “clusterprofiler” (Yu et al., 2012) package in R was used to perform GO function annotation and KEGG pathway enrichment analysis and visualized by the R package “GOplot” (Walter et al., 2015).

### Screen and Validation of Hub Genes

After choosing the interesting module, gene significance (GS) of >0.3 and module membership (MM) of >0.9 as the threshold for screening hub genes in the magenta module were set. The data sets TCGA were set as internal validation data sets, and the data sets GSE70770 and GSE141551 were selected as external validation data sets. All these were used to analyze the expression differences of hub genes with different Gleason scores and tumor stages. In addition, the expression of hub genes between PCa and adjacent tissues were confirmed by the data set GSE32571. One-way analysis of variance (ANOVA) or Student’s *t*-test was applied to measure the statistical significance of the calculated results. Furthermore, survival analysis was conducted for hub genes using R packages “survminer” and “survival.” To assess the diagnostic values of these genes, ROC curves were plotted and the area under the ROC curve (AUC) with the “pROC” R package was calculated (Robin et al., 2011).

### GSEA and GSVA

The R package “clusterprofiler” (Yu et al., 2012) was utilized to perform GSEA analysis of hub genes with TCGA-PRAD RNA-seq data. Moreover, the “GSVA” (Hänzelmann and Guinney, 2013) R package was used to find the pathways that are mostly associated with the hub genes. In this analysis, 498 PCa samples were divided into two groups (high vs. low expression) based on the median expression of each hub gene. *P* < 0.01 and log fold change of >0.15 were considered significant. The reference gene sets of GSEA and GSVA, which was called as “c2.cp.kegg.v6.2.symbols.gmt,” were downloaded from the Molecular Signatures Database^3^.

### Analysis of Tumor-Infiltrating Immune Cells

TIMER^4^ (Li et al., 2017) is an online tool that provides a comprehensive resource for systematical analysis of immune infiltrates across different types of cancers. We herein chose six tumor-infiltrating immune cells (B cells, CD4^+^ T cells, CD8^+^ T cells, macrophages, neutrophils, and dendritic cells) to investigate their correlation with the expression of selected hub genes.

### Genetic Alteration of Hub Genes

The cBioPortal for cancer genomics^5^, which is an open cancer genomics platform, analyzes, visualizes, and provides the service of downloading the data from multidimensional cancer genomics data sets (Cerami et al., 2012). The users can explore the genetic changes in different samples and genes. Here, the cBioPortal was utilized to investigate genetic alterations associated with hub genes.

### Related Small Molecule Drugs Screening

The CMap^6^ is an open resource that utilizes a variety of gene expression profiles for connecting small molecules, genes, and diseases (Lamb et al., 2006; Subramanian et al., 2017). First, small molecule drugs were used to process the human cells. After that, differential expression of genes in the magenta module was used to screen some molecule drugs that show high correlation with the disease. Finally, the enrichment score of each molecule drug was calculated, which ranged from –1 to 1. Also, a positive connective score suggests that a drug could induce signaling biology in a specific disease. In contrast, a negative connective score indicates that a drug could prevent signaling biology. To further investigate the association between small molecules and hub genes, a molecular docking simulation was performed on each drug dock with hub genes using Sybyl-X 2.1 software. It could help users to identify the molecule drugs against PCa.

## Results

### Screening of DEGs

**FIGURE 2:**
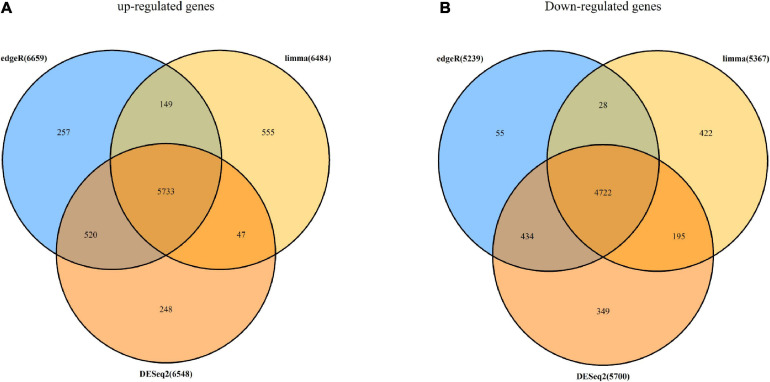
DEGs screened with limma, DESeq2, and edgeR algorithms. **(A)** Number of up-regulated DEGs identified with edgeR (light blue circle), limma (golden circle), and DESeq2 (deep orange circle) and overlapping DEGs (orange). **(B)** Number of down-regulated DEGs identified with edgeR (light blue circle), limma (golden circle), and DESeq2 (deep orange circle) and overlapping DEGs (orange).

The RNA-seq data of 498 tumor samples and 52 normal samples obtained from the TCGA data set underwent DEG analysis by three algorithms: limma (Ritchie et al., 2015), DESeq2 (Love et al., 2014), and edgeR (Robinson et al., 2009). Using the cutoff criteria of adj.*p*.value of <0.05, 11,851 DEGs were identified by limma, which included 6484 up-regulated DEGs and 5,367 down-regulated DEGs; 12,248 DEGs were screened by DESeq2, in which 6,548 were up-regulated and 5,700 were down-regulated; and 11,898 DEGs were selected by degeR, in which 6,659 DGEs were up-regulated and 5,239 DEGs were down-regulated. After that, 10,455 overlapping DEGs were chosen for further analysis by the above algorithms, which included 5,733 up-regulated DEGs and 4,722 down-regulated DEGs. A Venn diagram of DEGs is shown in Figure 2.

### WGCNA Analysis and Identification of Key Module

**FIGURE 3:**
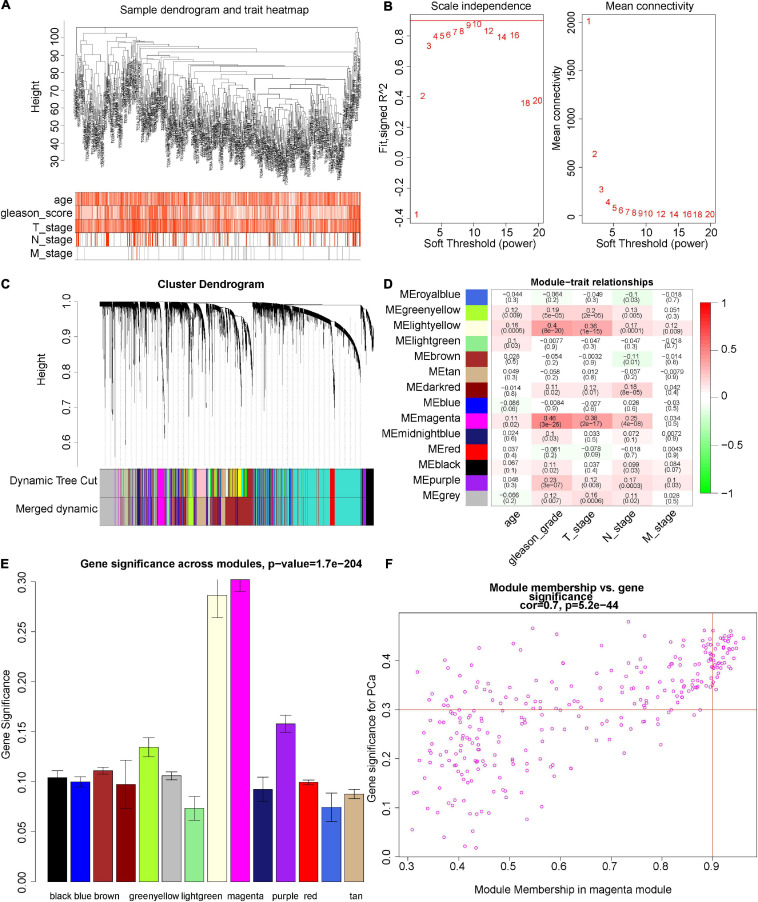
Construction of co-expression network. **(A)** Clustering dendrograms of genes. The clustering was based on 471 samples of TCGA-PRAD RNA-seq data. Color intensity varies positively with age, Gleason score, T stage, N stage, and M stage. **(B)** Analysis of the scale-free fit index (*R*^2^) and the mean connectivity with different soft-thresholding powers. *R*^2^ > 0.8, and the mean connectivity close to zero was considered as an appropriate soft threshold. Here, when we choose eight as our power, *R*^2^ = 0.87 (Supplementary Figure 1B). **(C)** Cluster dendrogram of all DEGs. Dendrograms are produced by average linkage hierarchical clustering of genes based on the topological overlap (Methods). The modules of co-expressed genes were assigned colors and numbers as indicated by the horizontal bar beneath each dendrogram (dynamic tree cut). In the clustering module of eigengenes, the module with height less than 0.25 was merged (Supplementary Figure 1C), and the results displayed in the Merged dynamics. **(D)** Overview of the modules generated by the WCGNA and their relationship with module eigengenes and clinical traits of PCa. The cells were color-coded by the correlation between module and clinical information according to the color legend on the right with red representing a strong positive correlation and green representing a strong negative correlation. **(E)** Module significance of each module, which is determined as the average absolute gene significance and errors measure for all genes in a given module associated with the Gleason score of PCa. **(F)** Scatterplot for ME magenta reveals the correlation between module membership (MM) and gene significance (GS). The dot indicates all genes within the magenta module. GS > 0.3 and MM > 0.9 are our criteria for selecting genes. The degree of association between MM and GS was assessed by Pearson correlation.

We herein constructed the weighted co-expression network by the “WGCNA” R package. The height of the sample clustering was defined as 108, when the 14 outlier samples were excluded from further analysis (Supplementary Figure 1A). Clinical sample information of data sets TCGA-PRAD, such as age, Gleason score, and TNM stage, were added below the resulting tree (Figure 3A). In the present study, the power of β was set as 8 [scale-free *R*^2^ = 0.87 (Supplementary Figure 1B)] to ensure a scale-free network (Figure 3B). By cutting the height of clustering of the module eigengenes as 0.25 (Supplementary Figure 1C) and setting the minimum cluster size of the gene dendrogram as 30, the genes with similar expression profiles were then classified into the same modules by the virtue of the DynamicTreeCut algorithm. Finally, 14 modules were clustered (Figure 3C). According to the heat map of module–trait correlations, the magenta module showed the most significant association with the clinical phenotypes, especially the Gleason score (*r* = 0.46, *p* = 3e–26), and T stage (*r* = 0.38, *p* = 2e–17; Figures 3D,E). Under the threshold of MM > 0.9 and GS > 0.3, 41 hub genes were obtained from the magenta modules: ANLN, ASF1B, AURKA, BUB1, CCNA2, CDC25C, CDCA5, CDCA8, CDK1, CDKN3, CENPA, CENPF, CENPI, CEP55, CKAP2L, DLGAP5, ERCC6L, ESPL1, EXO1, GTSE1, IQGAP3, KIF18B, KIF20A, KIF23, KIF2C, KIF4A, KIFC1, MELK, NCAPG, NEIL3, NEK2, NUF2, NUSAP1, PLK1, POLQ, RACGAP1, SGOL1, SKA3, SPAG5, TOP2A, and TPX2 (Figure 3F). Therefore, the magenta module was selected for further analysis.

### Magenta Module Function Annotation

**FIGURE 4:**
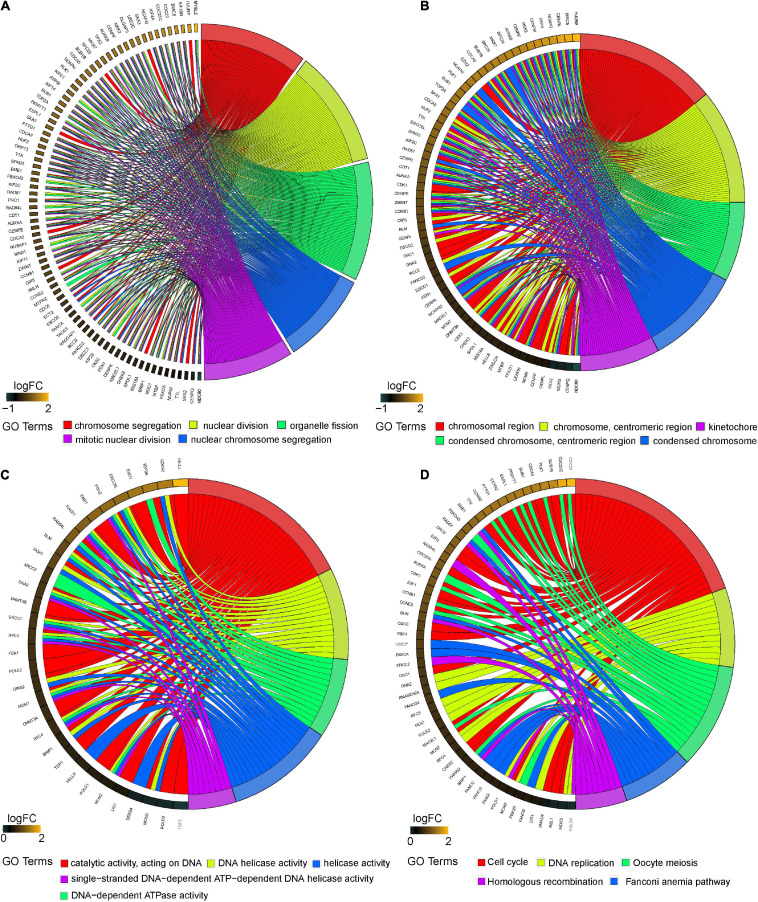
Functional enrichment analysis of the magenta module. Chord plot showing the relationship between all genes within the magenta module as identified by the WGCNA and GO terms and KEGG pathways. Outer ring displays log2 fold change (left) and GO terms and KEGG pathways (right). Chords connect the gene names with GO terms or KEGG pathway. **(A)** Top 5 significantly enriched biological process (BP) GO annotations. **(B)** Top 5 significantly enriched cellular component (CC) GO annotations. **(C)** Top 5 significantly enriched molecular function (MF) GO annotations. **(D)** Top 5 significantly enriched KEGG pathways. GO, Gene Ontology; KEGG, Kyoto Encyclopedia of Genes and Genomes.

The “clusterprofiler” R package was used to conduct GO and KEGG analyses to investigate the function of the magenta module. The GO analysis demonstrated that the biological process (BP) of the magenta module showed large correlation with “chromosome segregation,” “nuclear division,” “organelle fission,” “nuclear chromosome segregation,” and “mitotic nuclear division” (Figure 4A). The cellular component (CC) of the magenta module showed significant association with “chromosomal region”; “chromosome, centromeric”; “condensed chromosome, centromeric region”; “condensed chromosome”; and “kinetochore” (Figure 4B). The molecular function (MF) of the magenta module was mainly related to “catalytic activity, acting on DNA”; “DNA helicase activity”; “DNA-dependent ATPase activity”; “helicase activity”; and “single-stranded DNA-dependent ATP-dependent DNA helicase activity” (Figure 4C). In addition, the KEGG analysis revealed that “cell cycle,” “DNA replication,” “oocyte meiosis,” “fanconi anemia pathway,” and “homologous recombination” were mainly enriched in the magenta module (Figure 4D). This study illustrates that the genes within the magenta module were mainly involved in “cell cycle,” “DNA replication,” and “nuclear division.”

### Validation of the Expression of Real Hub Genes

**FIGURE 5:**
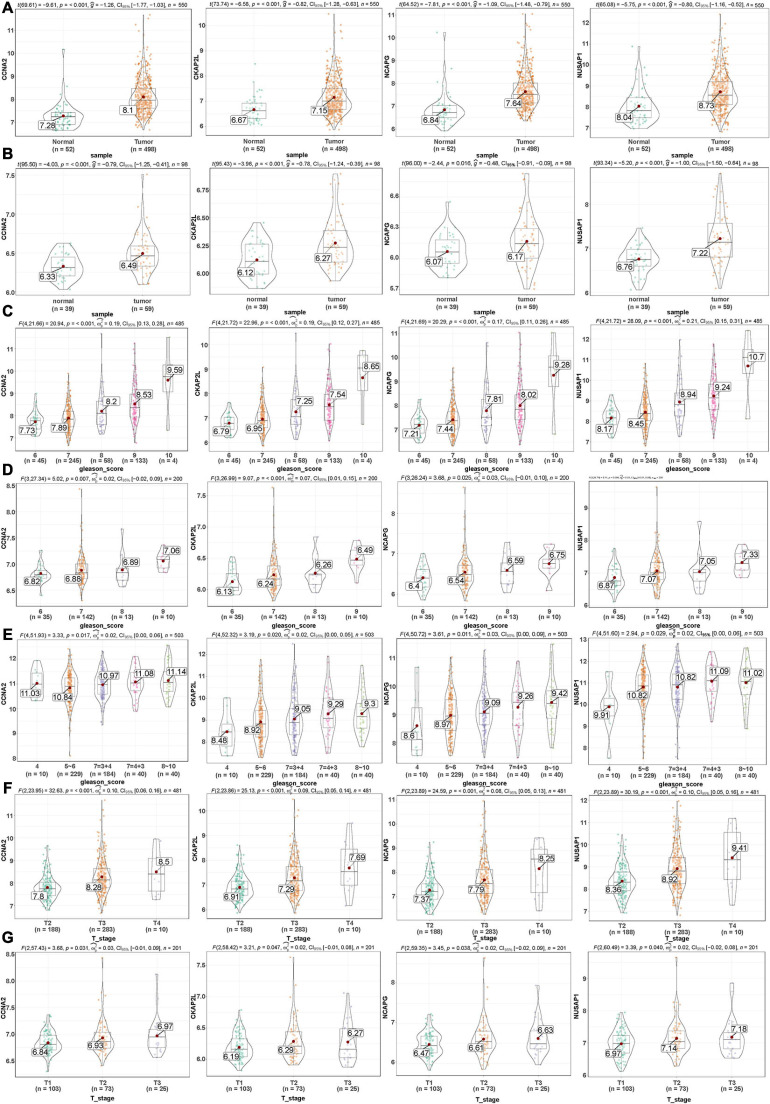
Validation of the gene expression levels of CCNA2, CKAP2L, NCAPG, and NUSAP1 by different data sets in various aspects. **(A)** Gene expression levels of CCNA2, CKAP2L, NCAPG, and NUSAP1 between PCa and normal samples in the TCGA-PRAD data set (internal validation data set). **(B)** CCNA2, CKAP2L, NCAPG, and NUSAP1 gene expression differences between PCa and normal samples in the GSE32571 data set (external validation data set). **(C)** Correlation of CCNA2, CKAP2L, NCAPG, and NUSAP1 with different Gleason scores (6, 7, 8, 9, and 10) in the TCGA-PRAD data set (internal validation data set). **(D)** Association of CCNA2, CKAP2L, NCAPG, and NUSAP1 with different Gleason scores (6, 7, 8, and 9) in the GSE70770 data set (external validation data set). **(E)** Expression of CCNA2, CKAP2L, NCAPG, and NUSAP1 in PCa samples with different Gleason scores (4, 5∼6, 7 = 3 + 4, 7 = 4 + 3, and 8∼10) in GSE141551 (external validation dataset). **(F)** Association between CCNA2, CKAP2L, NCAPG, and NUSAP1 expression and different T stage (T2, T3, and T4) in the TCGA-PRAD data set (internal validation data set). **(G)** Expression of CCNA2, CKAP2L, NCAPG, and NUSAP1 in PCa samples with diverse T stage (T1, T2, and T3) in GSE70770 (external validation data set). One-way analysis of variance (ANOVA) and Student’s *t*-test were utilized to calculate statistical differences in these data sets.

**FIGURE 6:**
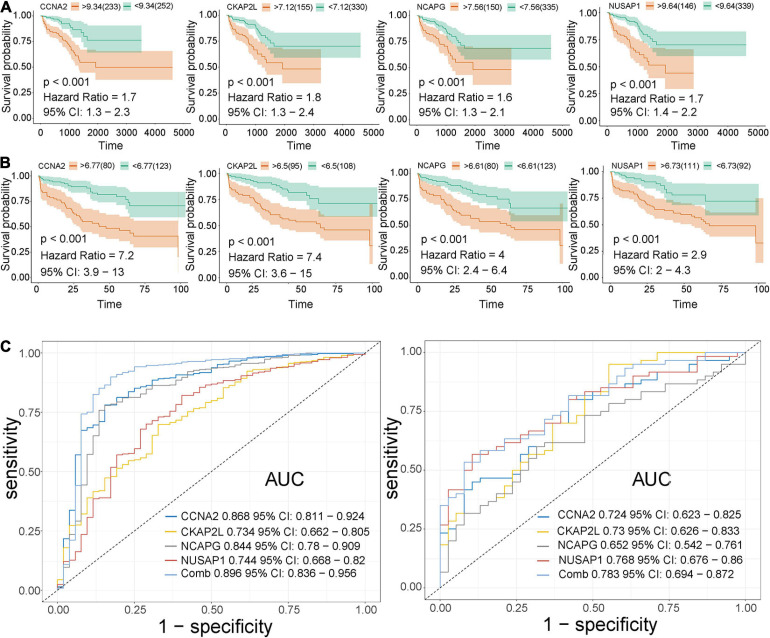
Validation of CCNA2, CKAP2L, NCAPG, and NUSAP1 in survival analysis and ROC curve. **(A)** Correlation between CCNA2, CKAP2L, NCAPG, and NUSAP1 expression and disease-free survival time based on the best separation in the TCGA-PRAD data set (internal validation data set). **(B)** Association between CCNA2, CKAP2L, NCAPG, and NUSAP1 expression and disease-free survival time based on the best-separation in the GSE70770 data set (external validation data set). Note: The yellow line denotes samples with highly expressed genes (above the best-separation value), and the green line represents samples with lowly expressed genes (below best-separation value). **(C)** Receiver operating characteristic (ROC) curves and area under the curve (AUC) statistics were conducted to evaluate the ability of hub genes to distinguish PCa from normal samples with specificity and sensitivity in the TCGA data set (left) and GSE32571 (right).

Here, among the 41 hub genes screened above, four genes (CCNA2, CKAP2L, NCAPG, and NUSAP1) were selected as our target hub genes and were seldom reported in PCa. After that, these genes were validated by the internal (TCGA) and external validation data sets (GSE32571, GSE70770, and GSE141551), respectively. First, the expression of these selected genes was verified between PCa samples and normal samples. Based on the TCGA data set, those four genes showed higher expression in the PCa tissues than normal tissues (Figure 5A), and the result is consistent with that in the validation data set (GSE32571; Figure 5B). Second, these genes were part of the magenta module, which showed significant association with the Gleason scores and the T stages of PCa. Thus, CCNA2, CKAP2L, NCAPG, and NUSAP1 were highly differentially expressed in PCa samples with different Gleason scores and T stages no matter whether in internal or external validation data sets. These results indicate that higher expression of hub genes showed correlation with higher Gleason scores and advanced T stages (Figures 5C–G). ANOVA and Student’s *t*-test were used to measure statistical significance of the calculated results. As for the prognosis, the survival analysis in the TCGA-PRAD data set was carried out to observe the disease-free survival of those four genes. This demonstrates that higher expression levels of those genes indicates poor disease-free survival (Figure 6A). The results are also consistent with that of the data set GSE70770 (Figure 6B). Furthermore, the ROC curve exhibited the excellent diagnostic and prognostic value of real hub genes in distinguishing between PCa and normal tissues (Figure 6C). In addition, immunohistochemistry staining based on the Human Protein Atlas database revealed that the protein levels of these four genes were significantly higher in tumor tissues compared with normal tissues. Similarly, compared with high-grade prostate tissues, the protein levels of hub genes were also significantly higher than low-grade prostate tissues (Supplementary Figures 2A–L).

### GSEA and GSVA Reveal the Function of Hub Genes

**FIGURE 7:**
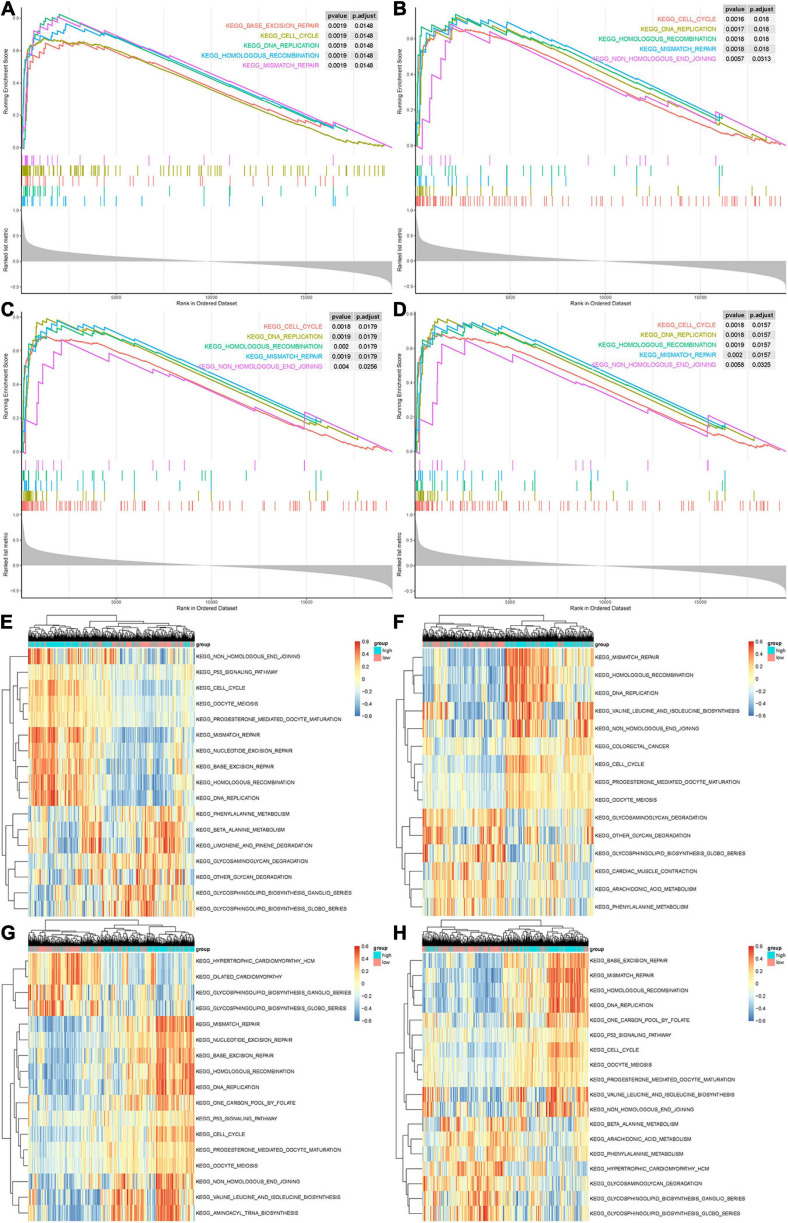
Gene set enrichment analysis (GSEA) and gene set variation analysis (GSVA) of hub genes. **(A–D)** The top 5 gene sets enriched in the high-expression group of single hub genes according to GSEA enrichment score. Each small bar represents a gene within the top 5 gene sets. It demonstrates the degree of correlation between genes in the top 5 gene sets and real hub genes. **(A)** CCNA2. **(B)** CKAP2L. **(C)** NCAPG. **(D)** NUSAP1. **(E–H)** Differentially expressed pathways of single hub genes were displayed based on the cluster heat map of GSVA. Scale on the right represents GSVA enrichment scores for individual gene sets. **(A)** CCNA2. **(B)** CKAP2L. **(C)** NCAPG. **(D)** NUSAP1. Signaling pathways with *P* < 0.01 and log fold change of >0.15 were considered significant.

Gene set enrichment analysis and GSVA analyses were conducted to further explore the potential functions of CCNA2, CKAP2L, NCAPG, and NUSAP1. As shown in Figures 7A–D, genes in high expression groups of CCNA2, CKAP2L, NCAPG, and NUSAP1 show enrichment in “cell cycle,” “DNA replication,” “homologous recombination,” and “mismatch repair” pathways. Subsequently, our analysis in the GSE32571 data set also produced similar results (Supplementary Tables 1–8). This evidence confirms that these real genes were highly connected with carcinoma proliferation. Furthermore, by applying GSEA analysis on the TCGA data, the previously reported “cell cycle,” “DNA replication,” “homologous recombination,” and “mismatch repair” pathways also exhibited a higher enrichment score in the high expression groups of these genes, further indicating their relationship with activation of proliferative processes (Figures 7E–H).

### Analysis of Tumor Purity and Immune Infiltration

**FIGURE 8:**
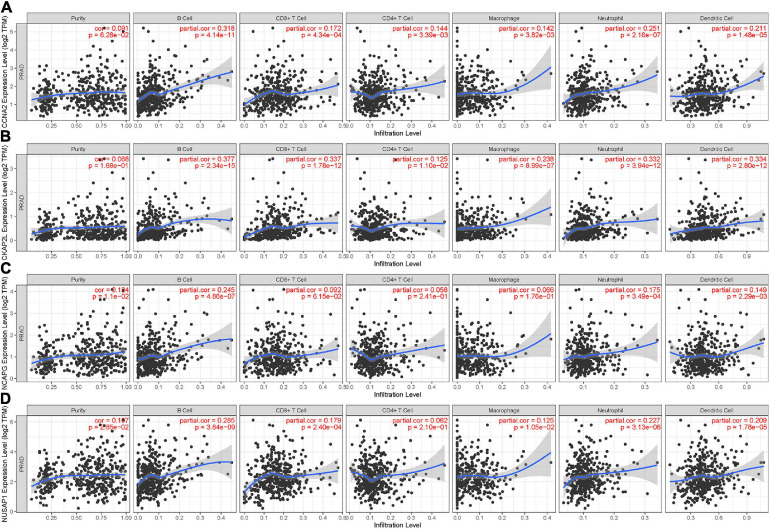
Relevancy of hub gene expression with immune infiltration level. **(A)** CCNA2. **(B)** CKAP2L. **(C)** NCAPG. **(D)** NUSAP1. *P* < 0.05 means statistical significance. Each dot represents a sample in the TCGA-PRAD data set.

The TIMER was used to assess immune cell infiltration levels of each sample based on the expression of hub genes. The expression of CCNA2 and CKAP2L showed positive association with infiltrating levels of B cells, CD8^+^ T cells, CD4^+^ T cells, neutrophils, macrophages, and dendritic cells, but were weakly positive with tumor purity (Figures 8A,B). Then, NCAPG expression showed a positive correlation with tumor purity and infiltration of B cells, neutrophils, and dendritic cells, and then, no or weak associations were observed between NCAPG and infiltration of CD8^+^ T cells, CD4^+^ T cells, and macrophages (Figure 8C). After that, NUSAP1 showed a positive relation with tumor purity and B cells, but the CD8^+^ T cells, neutrophils, macrophages, and dendritic cells showed no correlation with CD4^+^ T cells (Figure 8D). These results uncovered that these four genes were closely associated with the immune infiltration process of PCa, which might be a reason for them to become valid prognostic markers. Interestingly, in most of the samples, the tumor purity of these real hub genes was more than 0.5 (Figures 8A–D). This implies that the genes were mainly expressed in the tumor cells, proving the clinical diagnostic value of CCNA2, CKAP2L, NCAPG, and NUSAP1 again.

### Hub Genes Alterations in PCa

The PCa patient data in the TCGA database based on the cBioPortal database were utilized to analyze the alteration of the four key genes. As shown in Supplementary Figure 3A, CCNA2, CKAP2L, NCAPG, and NUSAP1 showed alteration of 6, 4, 4, and 6%, respectively, with mRNA high and deep deletion as the main types. Furthermore, among all the selected patients (499), four hub genes were altered in 60 (12%; Supplementary Figures 3A,B).

### Identification of Potentially Small Molecules

To identify candidate small molecules of PCa, the CMap database was used to screen out some small molecule drugs. According to the analysis of DEGs in the magenta module between tumor and normal samples, relevant small molecule drugs with high connection were identified. We herein set the criteria as number of instances (*n* > 10) and *P* value of <0.05, and then, 11 small molecule drugs were filtered (listed in Table 2). Both of them showed a negative correlation and had the potential to reverse the status of PCa. This analysis provides new ideas for the treatment of PCa.

**TABLE 2:** Results of CMap analysis.

	**CMap name**	**Mean**	***n***	**Enrichment**	***p***	**Specificity**
1	Tanespimycin	−0.426	62	−0.347	0	0.2437
2	Trichostatin A	−0.371	182	−0.333	0	0.4654
3	Tretinoin	−0.46	22	−0.402	0.001	0.0735
4	LY-294002	−0.341	61	−0.219	0.00475	0.5828
5	15-delta prostaglandin J2	−0.406	15	−0.418	0.00677	0.2181
6	Prochlorperazine	−0.428	16	−0.406	0.00678	0.1415
7	Vorinostat	−0.412	12	−0.436	0.01319	0.4956
8	Trifluoperazine	−0.342	16	−0.382	0.01322	0.2885
9	Sirolimus	−0.248	44	−0.233	0.01406	0.5548
10	Alvespimycin	−0.5	12	−0.416	0.02157	0.1884
11	Chlorpromazine	−0.285	19	−0.333	0.02216	0.1026

### Correlation Between Hub Genes and Compounds via Molecular Docking

A molecular docking study was carried out to investigate the association between molecules and hub genes. Using the SYBYL-X 2.1.1 software, the top 11 small molecule drugs identified by CMap analysis were docked with hub genes to obtain docking scores (Tables 3–6). Among the 11 top compounds, 15-delta prostaglandin J2 had the highest binding affinity to the four hub genes according to total score.

**TABLE 3:** Molecular docking of CCNA2.

**Name**	**Total_Score**	**Crash**	**Polar**
15-delta-prostaglandin-J2	9.5388	−2.1552	3.5693
Tanespimycin	7.4511	−3.0228	1.6463
Trifluoperazine	7.2148	−2.2088	0.8992
Prochlorperazine	6.984	−2.1011	1.2045
Tretinoin	6.9321	−1.8675	1.2962
Vorinostat	6.7712	−1.3365	3.5723
Alvespimycin	6.2191	−3.8744	3.7333
Trichostatin-A	5.8515	−1.1437	2.2318
Chlorpromazine	5.7682	−0.8164	2.7998
LY-294002	5.5317	−0.7601	2.3055
Sirolimus	4.622	−2.7804	2.2177

**TABLE 4:** Molecular docking of CKAP2L.

**Name**	**Total_Score**	**Crash**	**Polar**
delta-prostaglandin-J2	7.852	−1.5924	3.6404
Trifluoperazine	5.8955	−0.8018	1.7118
Vorinostat	5.2495	−1.185	2.2736
Prochlorperazine	4.8606	−0.732	1.483
Chlorpromazine	4.7681	−1.3495	1.7222
Alvespimycin	4.53	−1.6153	6.2844
Tretinoin	4.2592	−0.9624	1.4817
Trichostatin-A	3.7596	−1.3013	2.2535
LY-294002	3.6672	−0.858	0
Tanespimycin	2.5781	−1.3093	0.5352
Sirolimus	1.121	−1.8383	3.0711

**TABLE 5:** Molecular docking of NCAPG.

**Name**	**Total_Score**	**Crash**	**Polar**
Delta-prostaglandin-J2	6.7336	−1.3371	2.4941
Alvespimycin	5.1368	−1.699	0.9248
Tretinoin	4.9243	−1.004	2.6487
Vorinostat	4.747	−1.2161	4.1736
Trichostatin-A	4.6604	−1.0464	1.1727
Prochlorperazine	4.6098	−0.7403	1.6303
Trifluoperazine	4.408	−0.9068	1.7254
Chlorpromazine	3.6596	−0.6975	1.7134
Tanespimycin	3.2905	−0.9635	0.9676
LY-294002	3.2722	−1.0261	1.9495
Sirolimus	1.2984	−2.4359	1.1123

**TABLE 6:** Molecular docking of NUSAP1.

**Name**	**Total_Score**	**Crash**	**Polar**
Prochlorperazine	3.0117	−0.9845	0
Delta-prostaglandin-J2	2.8776	−0.9872	1.1538
Trichostatin-A	2.3572	−1.2679	0
Trifluoperazine	1.7683	−0.5994	0
Chlorpromazine	1.1286	−0.6207	0
Alvespimycin	1.0724	−0.9652	0
Tretinoin	0.8743	−0.7493	0
Vorinostat	0.6616	−0.838	0
LY-294002	0.2977	−1.2272	0
Tanespimycin	−0.3478	−2.735	0.0012
Sirolimus	−32.9961	−35.7588	0

## Discussion

Prostate cancer is a highly malignant tumor with complex pathogenesis. Recently, accumulated evidence has attempted to identify hub genes that play a significant role in the development and metastasis of PCa by virtue of microarray and RNA-seq (Varambally et al., 2005; Xu et al., 2018; Laufer-Amorim et al., 2019; Ma et al., 2019). However, to the best of our knowledge, few studies address interactions between genetic data and interrelated clinical information. In the current study, gene expression data sets and clinical data from the TCGA and GEO databases were used. Next, WGCNA was used to investigate gene co-expression modules that are correlated with the progression of PCa. After a range of rigorous screening, 41 hub genes (ANLN, ASF1B, AURKA, BUB1, CCNA2, CDC25C, CDCA5, CDCA8, CDK1, CDKN3, CENPA, CENPF, CENPI, CEP55, CKAP2L, DLGAP5, ERCC6L, ESPL1, EXO1, GTSE1, IQGAP3, KIF18B, KIF20A, KIF23, KIF2C, KIF4A, KIFC1, MELK, NCAPG, NEIL3, NEK2, NUF2, NUSAP1, PLK1, POLQ, RACGAP1, SGOL1, SKA3, SPAG5, TOP2A, and TPX2) that have a close relationship with Gleason scores and T stage were ultimately obtained. Most of these have been reported several times in PCa (Guo et al., 2006; Tamura et al., 2007; Vainio et al., 2012; Huang et al., 2017; Wilson et al., 2018). We herein selected four genes that are seldomly noticed in PCa (CCNA2, CKAP2L, NCAPG, and NUSAP1) as our target genes to further explore their function and value.

CCNA2, which is also known as cyclin A2, is a member of the cyclin family. CCNA2 is reported to regulate the cell cycle by binding to CDK or CDK2 to affect the G1/S phase and G2/M phase, respectively (Pagano et al., 1992). Published literature indicates that CCNA2 is overexpressed in multiple tumors, including breast, colorectal, gastric, pancreatic, and lung cancers (Gao et al., 2014; Gan et al., 2018; Zhang et al., 2018; Dong et al., 2019; Gao M. et al., 2020). As such, those cancers also showed significant association with histological grade, tumor stage, disease-free survival, and overall survival. In estrogen receptor + breast cancer, Gao et al. (2014) also found high levels of CCNA2, which showed correlation with tamoxifen treatment failure, in which it not only can be used as an independent prognostic factor, but also contributed to the monitoring of tamoxifen efficacy. Our results reveal that CCNA2 is up-regulated in PCa tissues when compared with normal tissues and that its expression is closely connected with Gleason scores, tumor stage in the TCGA database, and GEO data sets. Our study further corroborated Yang et al. (2020)’s research. All these suggest that CCNA2 plays an important role in PCa proliferation.

CKAP2L is a microtubule-associated protein that occurs during the mitotic phase and is involved in neural progenitor cell division (Yumoto et al., 2013). Specifically speaking, down-regulation of CKAP2L gives rise to separation between the multipolar spindles and the chromosome in the neural progenitor cells. In addition, Jakobsen et al. (2011) identified CKAP2L as part and parcel of the centrosome situated in the spindle, i.e., in the midbody and the spindle pole. According to the published literature, little is known about the role of CKAP2L in tumor development and proliferation. A recent paper reports high expression of CKAP2L, which induces the invasion of lung adenocarcinoma by the MAPK signaling pathway, and shows correlation with poor prognosis (Xiong et al., 2019). Furthermore, CKAP2 acts as a significant paralog of CKAP2L, and the oncogenic nature and overexpression of this gene is revealed in PCa (Yu et al., 2015), ovarian cancer (Gao et al., 2017), and glioma (Wang et al., 2018). In this regard, as a novel mitotic spindle protein, we speculate that it might regulate cancer progression by taking part in the polymerization of microtubules and then affecting cell mitosis.

NCAPG is a key component of the condensin complex and is highly correlated with the condensation and stabilization of chromosomes during mitosis and meiosis (Murphy and Sarge, 2008). It is located on human chromosome band 4p15.32 and encoded by the NY-MEL-3 gene (Jäger et al., 2000). To date, previous study shows the involvement of NCAPG in hepatocellular carcinoma and breast, lung, and ovarian cancers (Cao and Zhang, 2016; Cava et al., 2018). In hepatocellular carcinoma, Wang (Wang et al., 2019) verifies that depletion of NCAPG contributes to hepatocellular carcinoma cell cycle arrest at the S phase and induces apoptosis. In addition, they also find that NCAPG could serve as a promoter of invasion and metastasis of liver cancer. In PCa, our study demonstrates that the expression of NCAPG shows significant association with tumor stage and disease-free survival, and this is in agreement with the reports put forwarded by Arai (Arai et al., 2018) in castration-resistant PCa clinical specimens.

As a cell-cycle related protein, NUSAP1 plays an important role in mitotic progression, spindle formation, and stability. In 2003, Raemaekers et al. (2003) first found it as a novel 55-kD vertebrate protein and showed selective expression in proliferative cells. There is much evidence to indicate that NUSAP1 is closely associated with apoptosis, proliferation, and metastasis. For instance, in cervical cancer (Li et al., 2019), NUSAP1 is shown to bound to the SUMO-E3 ligase Ran binding protein 2 (RanBP2) to induce the sumoylation of TCF4, thereby enhancing the Wnt/β signaling pathway and inducing tumor metastasis. Similarly, a high NUSAP1 expression level facilitated the development of bladder cancer by regulating epithelial-mesenchymal transition of bladder cancer via the TGF-β signaling pathway (Gao S. et al., 2020). In addition, Liu (Liu et al., 2018) reports that the mRNA and protein levels of NUSAP1 were more highly expressed in colon cancer than in paired non-cancerous adjacent tissues. These results also support the studies of Gordon et al. (2017).

We herein also provide substantial evidence in support of the diagnostic and prognostic values of CCNA2, CKAP2L, NCAPG, and NUSAP1, which range from internal to external validation data sets. The genes CCNA2, CKAP2L, NCAPG, and NUSAP1 were not only significantly up-regulated in PCa tissues, but also positively associated with higher Gleason score and tumor stage, implicating significant contributions to the pathogenesis of PCa. In addition, survival analysis shows that high expression of hub genes is related to a shorter disease-free survival in PCa. Furthermore, ROC curve analysis of the four hub genes was conducted, and the protein expression levels were analyzed based on the HPA database, which provides evidence that these four genes have a high diagnostic and prognostic value in PCa.

It is well known that tumors are composed of not only tumor cells, but also of stroma and immune cells. Hence, several immune cells (B cells, CD4^+^ T cells, CD8^+^ T cells, macrophages, neutrophils, and dendritic cells) were assessed in TIMER. These hub genes show positive correlation with the majority of the above immune cells. Unexpectedly, the tumor purity of hub genes in most of the samples was more than 0.5. Based on these findings, we suspect that CCNA2, CKAP2L, NCAPG, and NUSAP1 might be mainly expressed in PCa cells. GSEA and GSVA were further utilized to explore the biological function. Cell cycle and cell cycle–related pathways, such as DNA replication, mismatch repair, and recombination, were mainly observed as enriched pathways, indicating that they can lead to tumor proliferation.

In addition, the CMap database was utilized to identify some small molecule drugs with potential therapeutic benefits against PCa. Most of these have been previously documented to have anticancer effects in various cancer types. Among the molecule drugs, vorinostat (Schneider et al., 2012), alvespimycin (Pacey et al., 2011), and tretinoin (Ueda et al., 2020) have undergone phase I clinical trials in cancer patients. In the early 1990s, some experimental evidence revealed that tanespimycin had antitumor activity in various human-derived tumor cell lines (Erlichman, 2009). Trichostatin A, which is a histone deacetylase inhibitor, had an underlying therapeutic effect in diverse cancer cells when combined with radiotherapy or chemotherapy. Trifluoperazine was originally an antipsychotic drug, whereas some recent literature report that it could inhibit cancer cell proliferation, such as hepatocellular carcinoma (Jiang et al., 2017), lung cancer (Yeh et al., 2012), and glioblastoma (Pinheiro et al., 2017). Moreover, the anticancer effects of other small molecule drugs are also mentioned by the researchers. Molecular docking analysis demonstrates that 15-delta prostaglandin J2 had the highest binding affinity to four hub genes in 11 small molecule drugs. Prostaglandin J2, a potent activator of PPAR-γ (Kliewer et al., 1995), is shown to inhibit serum-stimulated cell proliferation in vascular smooth muscle cells. Thus, we speculated that 15-delta prostaglandin J2 possessed the antitumor effects via inhibited serum-stimulated cell proliferation. In brief, this information remains to be beneficial for the development of targeted therapy in PCa.

However, there are some deficiencies that should be acknowledged. First, this was a retrospective study rather than a prospective cohort study, and all data in this study were acquired from the open available databases. In addition, further research, including *in vivo* and *in vitro* experiments are needed to elucidate the potential molecular mechanisms of how the four genes impact on Gleason score and tumor stage.

Taken together, CCNA2, CKAP2L, NCAPG, and NUSAP1 were successfully identified as our candidate genes and small molecular drugs with the potential to treat PCa. Of clinical significance, the four genes might serve as potential biomarkers for PCa, and these molecular drugs might provide a new avenue for the treatment of PCa.

## Data Availability Statement

Publicly available datasets were analyzed in this study. This data can be found here: https://www.cancer.gov/ The Cancer Genome Altas (TCGA), https://www.ncbi.nlm.nih.gov/ Gene Expression Omnibus (GEO) GSE32571, https://www.ncbi.nlm.nih.gov/ Gene Expression Omnibus (GEO) GSE70770, and https://www.ncbi.nlm.nih.gov/ Gene Expression Omnibus (GEO) GSEGSE141551.

## Author Contributions

All authors listed have made a substantial, direct and intellectual contribution to the work, and approved it for publication.

## Conflict of Interest

The authors declare that the research was conducted in the absence of any commercial or financial relationships that could be construed as a potential conflict of interest.

## Footnotes

1 https://www.cancer.gov/

2 https://www.ncbi.nlm.nih.gov/

3 http://software.broadinstitute.org/gsea/msigdb/index.jsp

4 https://cistrome.shinyapps.io/timer/

5 http://www.cbioportal.org/

6 http://www.broadinstitute.org/CMap/
