# Supplementary material for: Four Novel Prognostic Genes Related to Prostate Cancer Identified Using Co-expression Structure Network Analysis
Source: Front Genet. 2021 Apr 1;12:584164. doi: 10.3389/fgene.2021.584164 (PMC8078837; doi:10.3389/fgene.2021.584164)
Supplement: Supplementary file 1 [file Data_Sheet_1.docx]

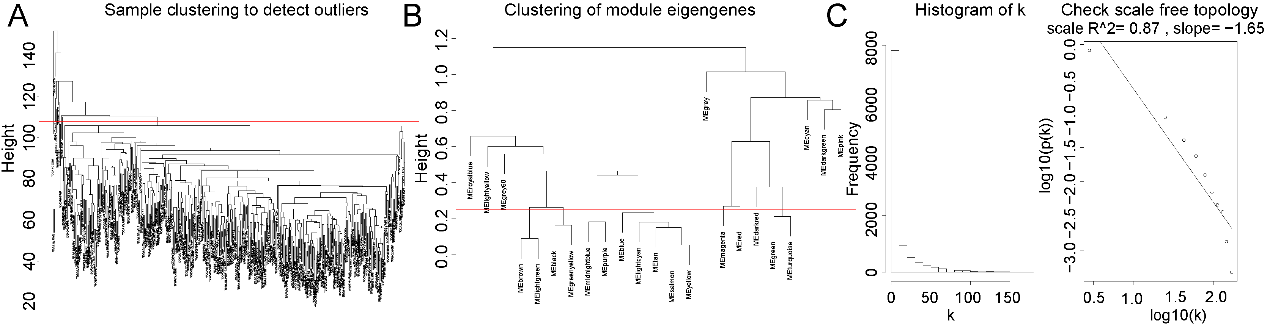


**Supplementary Figure1 |**

**WGCNA analysis**

(A) clustering of samples to detect outliers. The red line represents the height of sample clustering, which set as 108.

1. The cluster dendrogram of module eigengenes. The red line indicates cut height (0.25)
2. The left panel is histogram of connectivity distribution when β= 8, the right picture is analysis of the mean connectivity for various soft-thresholding powers.

**
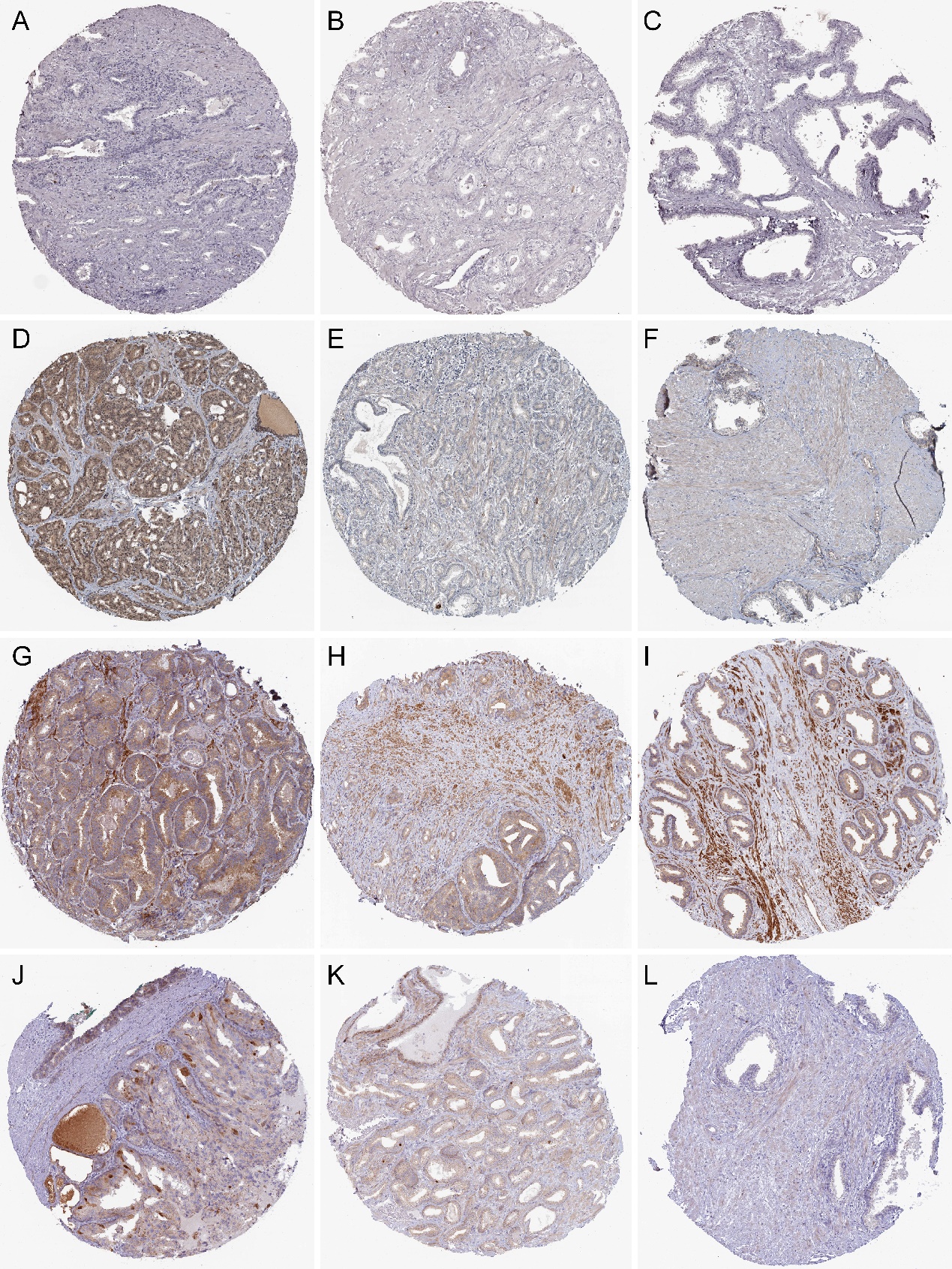
**

**Supplementary Figure2 |**

**Immunohistochemistry of the 4 key genes based on the Human Protein Atlas (HPA) database.**

(A) Protein levels of CCNA2 in normal prostate tissue (Staining: Not detected; Intensity: Negative; Quantity: None). (B) Protein levels of CCNA2 in low-grade prostate tissue (Staining: Low; Intensity: Moderate; Quantity: <25%). (C) Protein levels of CCNA2 in high-grade prostate tissue (Staining: Medium; Intensity: Strong; Quantity: <25%). (D) Protein levels of CKAP2L in normal prostate tissue (Staining: Not detected; Intensity: Weak; Quantity: <25%). (E) Protein levels of CKAP2L in low-grade prostate tissue (Staining: Low; Intensity: Weak; Quantity: 75%~25%). (F) Protein levels of CKAP2L in high-grade prostate tissue (Staining: Medium; Intensity: Moderate; Quantity: >75%). (G) Protein levels of NCAPG in normal prostate tissue (Staining: Medium; Intensity: Moderate; Quantity: 75%~25%). (H) Protein levels of NCAPG in low-grade prostate tissue (Staining: Medium; Intensity: Moderate; Quantity: >75%). (I) Protein levels of NCAPG in high-grade prostate tissue (Staining: Medium; Intensity: Moderate; Quantity: >75%). (J) Protein levels of NUSAP1 in normal prostate tissue (Staining: Medium; Intensity: Moderate; Quantity: 75%~25%). (K) Protein levels of NUSAP1 in low-grade prostate tissue (Staining: Low; Intensity: Weak; Quantity: 75%~25%). (L) Protein levels of NUSAP1 in high-grade prostate tissue (Staining: Medium; Intensity: Strong; Quantity: <25%).

**
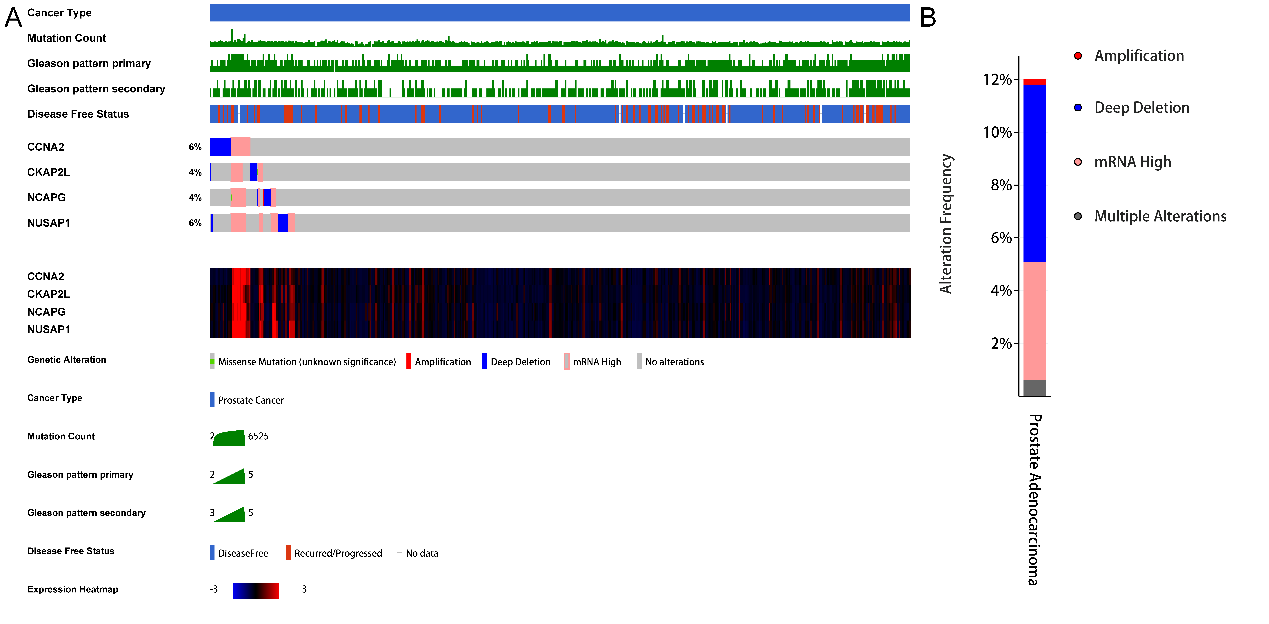
**

**Supplementary Figure3 |**

**Genetic alteration of hub genes**

(A) Summary of the frequency of genetic alterations in CCNA2, CKAP2L, NCAPG, and NUSAP1 in 499 samples. Meanwhile, the information such as heatmap of hub genes, mutation count, Gleason pattern and disease-free survival (DFS) of each sample was also showed.

(B) The total alteration frequency of 4 hub genes.
